# Establishment of a Prognostic Model Using Immune-Related Genes in Patients With Hepatocellular Carcinoma

**DOI:** 10.3389/fgene.2020.00055

**Published:** 2020-02-25

**Authors:** Wen-Jie Wang, Han Wang, Ting-Yan Hua, Wei Song, Jie Zhu, Jing-Jing Wang, Yue-Qing Huang, Zhi-Liang Ding

**Affiliations:** ^1^ Department of Radio-Oncology, The Affiliated Suzhou Hospital of Nanjing Medical University, Suzhou, China; ^2^ Department of Oncology, Jining Cancer Hospital, Jining, China; ^3^ Department of Gastroenterology, The Affiliated Suzhou Hospital of Nanjing Medical University, Suzhou, China; ^4^ Department of Gastrointestinal Surgery II, Renmin Hospital of Wuhan University, Wuhan, China; ^5^ Department of Oncology, Changzhou Traditional Chinese Medical Hospital, Changzhou, China; ^6^ Department of Oncology, Taizhou Hospital of Traditional Chinese Medicine, Taizhou, China; ^7^ Department of General Medicine, The Affiliated Suzhou Hospital of Nanjing Medical University, Suzhou, China; ^8^ Department of Neurosurgery, The Affiliated Suzhou Hospital of Nanjing Medical University, Suzhou, China

**Keywords:** hepatocellular carcinoma, immune-related genes, ceRNA network, prognostic model, The Cancer Genome Atlas (TCGA)

## Abstract

Hepatocellular carcinoma (HCC) is one of the most prevalent neoplasms worldwide, particularly in China. Immune-related genes (IRGs) and immune infiltrating lymphocytes play specific roles in tumor growth. Considering how important immunotherapy has become for HCC treatment in the past decade, our objective was to establish a prognostic model by screening survival-related IRGs in patients with HCC. Using *edgeR*, we identified differentially expressed IRGs (DEIRGs), DEmiRNAs, and DElncRNAs. Functional enrichment analysis of DEIRGs was performed to investigate the biological functions of IRGs *via* gene ontology annotation and Kyoto Encyclopedia of Genes and Genomes (KEGG) pathway analyses. Protein-protein interaction and competing endogenous RNA networks were established using Cytoscape. Survival-associated IRGs were selected *via* univariate COX regression analysis, a The Cancer Genome Atlas (TCGA) prognostic model and GSE76427 validation model were developed using multivariate COX regression analysis test by AIC (Akaike Information Criterion). We identified 116 DEIRGs in patients with HCC; the “cytokine-cytokine receptor interaction” pathway was found to be the most enriched pathway. *Via* the prognostic model helped us classify patients into high- and low-risk score groups based on overall survival (OS); high risk score was associated with worse OS, and a positive correlation was observed between the prognostic model and immune cell infiltration. To summarize, we established a prognostic model using survival-related IRGs that provides sufficient information for prognosis prediction and immunotherapy of patients with HCC.

## Introduction

Primary liver cancer is one of the most common and lethal cancers in China, with a 5-year survival rate of <5%. It can be classified into hepatocellular carcinoma (HCC) and cholangiocarcinoma (Chen et al., 2016; Bray et al., 2018). Recent studies have reported that the incidence and mortality of liver cancer have declined in China, but considering the large population, a large number of new cases are still reported each year (Chen et al., 2016).

HCC is usually associated with chronic hepatitis and cirrhosis attributable to hepatitis B or hepatitis C virus (HBV and HCV, respectively) infections (Marrero et al., 2018). HCC caused by chronic hepatitis is associated with complex mechanisms; for example, hepatitis virus infection can disrupt the immune system, leading to immune cell infiltration and cytokine secretion (Wang et al., 2013).

Patients with HCC show high mortality rates, which can be attributed to immune evasion, drug tolerance, and distant metastasis (Nagao et al., 2000; Chi et al., 2016). In the past decade, immunotherapy has emerged as a powerful modality to treat various conditions, considering that our understanding of how the immune system functions has substantially increased. Immunological checkpoint inhibitors such as pembrolizumab and nivolumab appear promising for treating patients with advanced liver cancer (El-Khoueiry et al., 2017; Zhu et al., 2018). Several studies have reported that immune cell infiltration is associated with HCC prognosis (Zhou et al., 2016; Zhou et al., 2019). Some studies have moreover described the correlation between immune-related genes (IRGs) and tumor prognosis, but few are based on HCC (Li B. et al., 2017; Lin et al., 2019).

In the present study, our objective was to establish a prognostic model by screening survival-related IRGs. We analyzed the correlation between the model and immune infiltrating cells with the aim of providing sufficient information for prognosis prediction and immunotherapy of patients with HCC.

## Material and Methods

### Subjects

A total of 10 pairs of HCC tissues and the corresponding adjacent normal tissues were obtained from HCC patients undergoing surgery at the Affiliated Suzhou Hospital of Nanjing Medical University (China). None of the patients had received chemotherapy or radiotherapy prior to surgery. Before surgery, written informed consent was collected from HCC patients. This work was approved by the Medical Ethics Committees of the Affiliated Suzhou Hospital of Nanjing Medical University.

### Data Acquisition and Processing

RNA-sequencing and clinical data of patients with HCC were obtained from The Cancer Genome Atlas (TCGA) data portal (https://portal.gdc.cancer.gov/) and Gene Expression Omnibus (GEO) data portal (https://www.ncbi.nlm.nih.gov/geo/). A total of 374 HCC and 50 adjacent non-cancer liver tissues were included in TCGA. GSE76427 was included 115 HCC patients. The TCGA hepatocellular carcinoma patients' clinical feathers were list in **Table 1**. The GSE76427 hepatocellular carcinoma patients' clinical feathers were list in **Table S1**. A list of IRGs was downloaded from the Immunology Database and Analysis Portal system (Bhattacharya et al., 2014).

**Table 1:** The clinical features of TCGA hepatocellular carcinoma patients.

Clinical features		
Age (years)	media	73
	Rage	18-90
	Numbers of patients (n=374)	Numbers of patients (%)
Gender	Female	120 (32.09)
	Male	254 (67.91)
Grade	G1	55 (14.71)
	G2	179 (47.86)
	G3	136 (36.36)
	unknown	4 (1.07)
T stage	T1	185 (49.47)
	T2	94 (24.13)
	T3	81 (21.66)
	T4	13 (4.74)
N stage	0	257 (68.72)
	1	5 (1.37)
	unknown	113 (29.91)
M stage	0	364 (97.32)
	1	5 (1.34)
	unknown	5 (1.34)
AJCC stage	I	175 (46.78)
	II	86 (25.27)
	III	86 (25.27)
	IV	5 (1.34)
	unknown	5 (1.34)

AJCC, American Joint Committee on Cancer; M, metastasis, N, node; T, tumor; TCGA, The Cancer Genome Atlas.

### Identification of Differentially Expressed Genes (DEGs), Differentially Expressed IRGs (DEIRGs), and Survival-Associated IRGs

DEGs were identified using the *edgeR* package in the R statistical environment (http://bioconductor.org/packages/edgeR/) (R Development Core Team, Vienna, Austria) and then further analyzed. |Log_2_ fold change (FC)| > 2.0 and false discovery rate (FDR) adjusted to *P* < 0.05 were set as the thresholds (Robinson et al., 2010). A list of immune-related genes was downloaded from the Immunology Database and Analysis Portal (ImmPort, https://www.immport.org/shared/genelists), which shares basic immunological data for cancer research. The DEIRGs were obtained by intersect the previously acquired DGEs list with the immune-related genes list. In addition, we generated volcano and heat maps of DEGs and DEIRGs using the *gplots* and *heatmap* packages in the *R* platform. Survival-associated DEIRGs were selected *via* univariate Cox regression analysis, which was performed using the *survival* package in the R platform.

### Functional Enrichment Analysis

To understand the biological mechanisms underlying IRGs in the prognostic model, gene ontology (GO) annotation and Kyoto Encyclopedia of Genes and Genomes (KEGG) pathway analyses were performed using the DAVID (https://david.ncifcrf.gov/) online tool and *ClusterProfiler*, which is an R package for functional classification and enrichment of gene clusters using hypergeometric distribution (Dennis et al., 2003). The results of GO annotation and KEGG pathway analyses were visualized using the *GOplot* package in R. We constructed a visualized network using Cytoscape 3.6.1 (National Resource for Network Biology, USA). GO annotation and KEGG pathway analyses were based on the threshold of *P* < 0.05.

### Constructing a Protein-Protein Interaction (PPI) Network

To clarify the potential relationships of DEmRNAs, a protein-protein interaction (PPI) network was constructed using the Search Tool for the Retrieval of Interacting Genes/Proteins (STRING) 10.5 (https://string-db.org/cgi/input.pl) and visualized by Cytoscape. We then used CytoHubba to identify hub genes.

### Construction of the Competing Endogenous RNA (ceRNA) Network

To further analyze the potential targets of key genes, we established a competing endogenous RNA (ceRNA) network. First, through *edgeR* package in the R statistical environment, we identified differentially expression miRNA (DEmiRNA) and differentially expression LncRNA (DELncRNA). |Log_2_ fold change (FC)| > 2.0 and FDR adjusted to *P*< 0.05 were set as the thresholds. Using miRcode (http://www.mircode.org/), we predict the DEmiRNA related DELncRNA in the network. Then through miRDB, miRTarBase, and TargetScan databases, we identified potential target genes for DEmiRNA. Intersecting these target genes with DEIRGs, we obtained the mRNA in the network. Based on miRNA-LncRNA and miRNA-mRNA, we established a ceRNA network, which was visualized using Cytoscape.

### Development of the IRG Prognostic Model (IRGPM) and Validation Model

Survival-associated IRGs were selected *via* univariate Cox regression analysis using the *survival* package in the R platform. Patients with HCC were divided into high- and low-risk groups using the median risk score as the cut-off *via* multivariate Cox regression analysis test by AIC (Akaike Information Criterion). To verify the feasibility of the prognostic model, we also divided GSE76427 patients into two groups according to the median risk score. The survival of the two groups was analyzed by KM curve. The risk score calculation formula for all patients is as follows

$$SurvivalRiskScore\;(SRS)=\sum_{i=1}^{k}\left(C_{i}\times V_{i}\right)$$

In the formula, *k* represents the number of mRNA, *C_i_* represents the coefficient of mRNA in multivariate Cox regression analysis, and *V_i_* represents the expression level of mRNA.

### Relationship Between IRGPM and Immune Cell Infiltration

We used the Tumor Immune Estimation Resource (TIMER) web server to infer the abundance of tumor infiltrating immune cells (Li T. et al., 2017). TIMER re-analyzes gene expression data and the database includes 10,897 samples across 32 cancer types from TCGA to estimate the abundance of six subtypes of tumor-infiltrating immune cells, including CD4 T cells, CD8 T cells, B cells, macrophages, dendritic cells (DCs), and neutrophils. Thus, it can be effectively used to determine the relationship between immune cell infiltration and other parameters. We downloaded data pertaining to immune cell infiltration levels among patients with HCC and assessed the association between IRGPM and immune cell infiltration.

### Quantitative Real-Time PCR (qRT-PCR)

Total RNA was obtained from 10 patients with HCC using TRIzol reagent (Invitrogen, Carlsbad, USA), and then reverse transcribed with the First Strand cDNA synthesis kit (New England Biolabs, Beijing, China). We performed amplifications with a SYBR Green PCR kit (Applied Biological Materials, Richmond, Canada) according to the manufacturer's instructions on Applied Biosystems 7500Real-Time PCR System (Applied Biosystems, Foster city, USA). The expression of RNA was normalized against GAPDH using the 2-^ΔΔCt^ method. The PCR primers used are shown in **Table 2**. Three separate experiments were performed.

**Table 2:** Primer sequences for qRT-PCR.

Primer	Seqence 5'-3'
IL11 forward	GTGGCCAAGATACAGCTGTCGC
IL11 reverse	GGTAGGACAGTAGGTCCGCTC
IL17 forward	AGA GAT ATC CCT CTG TGATC
IL17 reverse	TAC CCC AAA GTT ATC TCA GG
SPP1 forward	TCACCTGTGCCATACCAGTT
SPP1 reverse	TGTGGTCATGGCTTTCGTTG
MAPT forward	AAGATCGGCTCCACTGAGAA
MAPT reverse	ATGAGCCACACTTGGAGGTC
BIRC5 forward	CGCATCTCTACATTCAAG
BIRC5 reverse	ATGTTCCTCTCTCGTGAT
miR-204 forward	ACACTCCAGCTGGGTTCCCTTTGTCATCC
miR-204 reverse	CTCAACTGGTGTCGTGGA
SNHG12 forward	TCTGGTGATCGAGGACTTCC
SNHG12 reverse	ACCTCCTCAGTATCACACACT
GAPDH forward	CAACGAATTTGGCTACAGCA
GAPDH reverse	AGGGGTCTACATGGCAACTG

qRT-PCR, Quantitative real-time PCR.

### Statistical Analysis

Survival analysis of data related to patients in the model was performed using the *survival* package in the R platform. Survival curves were generated using the Kaplan-Meier method, and the log-rank test was used to compare differences between the two groups. The area under the receiver operating characteristic (ROC) curve was calculated using the *survival ROC* package to validate the performance of the prognostic signature (Heagerty et al., 2000). HCC and normal tissue data are reported as means±SD and were analyzed by GraphPad Prism 8.0 software (GraphPad Software Inc., USA). The significant difference between groups was evaluated using Student's t-test for a single comparison or one-way analysis of variance (ANOVA) with Bonferroni post-hoc test for multiple comparisons. Analysis items with *P*< 0.05 were considered statistically significant.

## Results

### Identification DEGs

**Figure 1:**
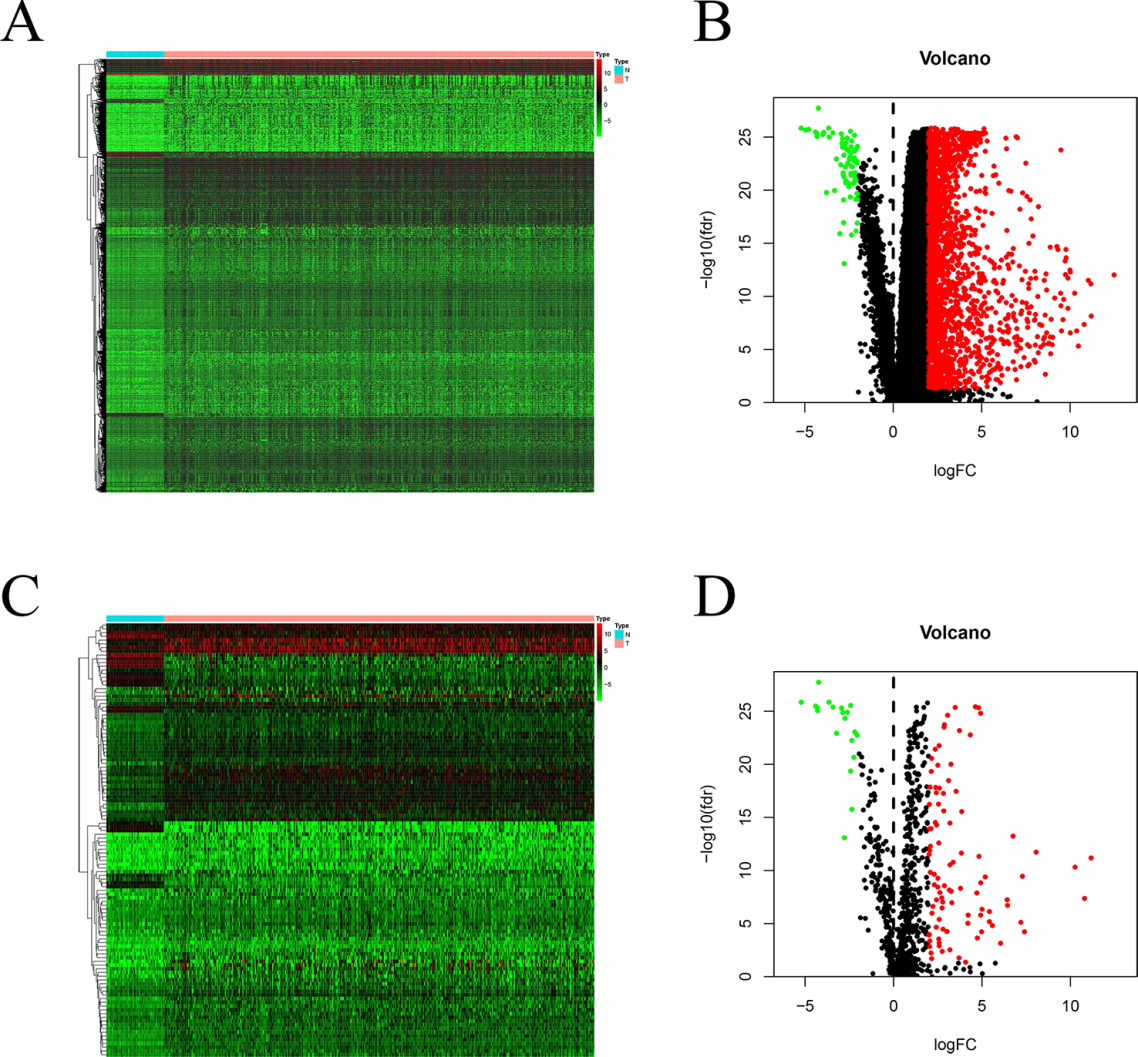
DEGs and DEIRGs in patients with HCC: Heatmap **(A)** and volcano plot **(B)** of DEGs in HCC and adjacent non-cancer liver tissues. Heatmap **(C)** and volcano plot **(D)** of DEIRGs in HCC and adjacent non-cancer liver tissues.

Using *edgeR* v3.53, we identified 2,068 DEGs among patients with HCC; 1,991 and 77 were up- and downregulated, respectively, with the thresholds of |log_2_ FC| > 2.0 and adjusted *P* < 0.05 (**Figures 1A, B**). Upon further comparison with the list of IRGs, we identified 116 DEIRGs; 96 and 20 were up- and downregulated, respectively, with the same thresholds (**Figures 1C, D**).

### Construction of the Immune-Related Genes Prognostic Model (IRGPM) and Validation Model

**Figure 2:**
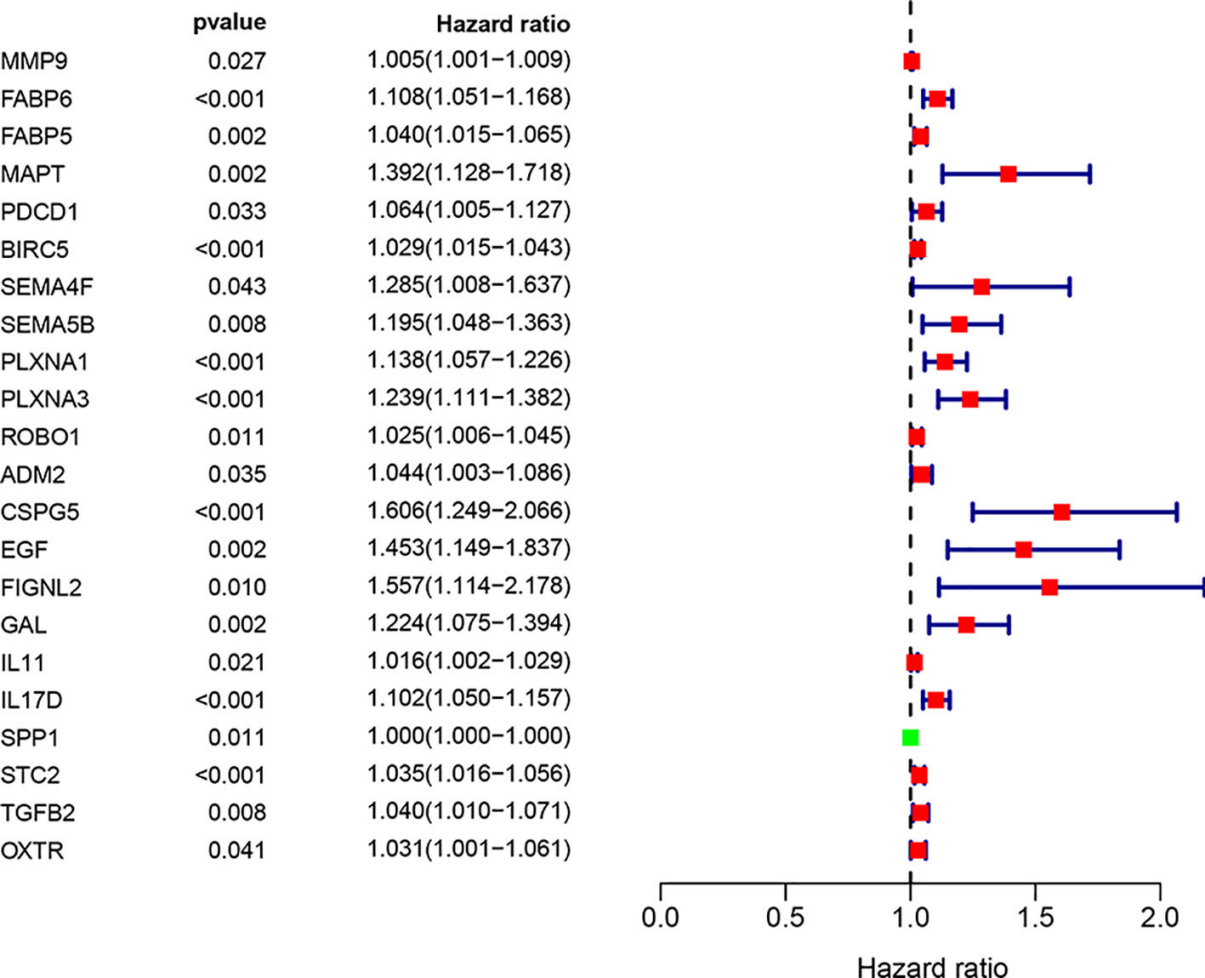
Prognostic values of survival-associated IRGs: Forest plot of survival-associated IRGs.

**Figure 3:**
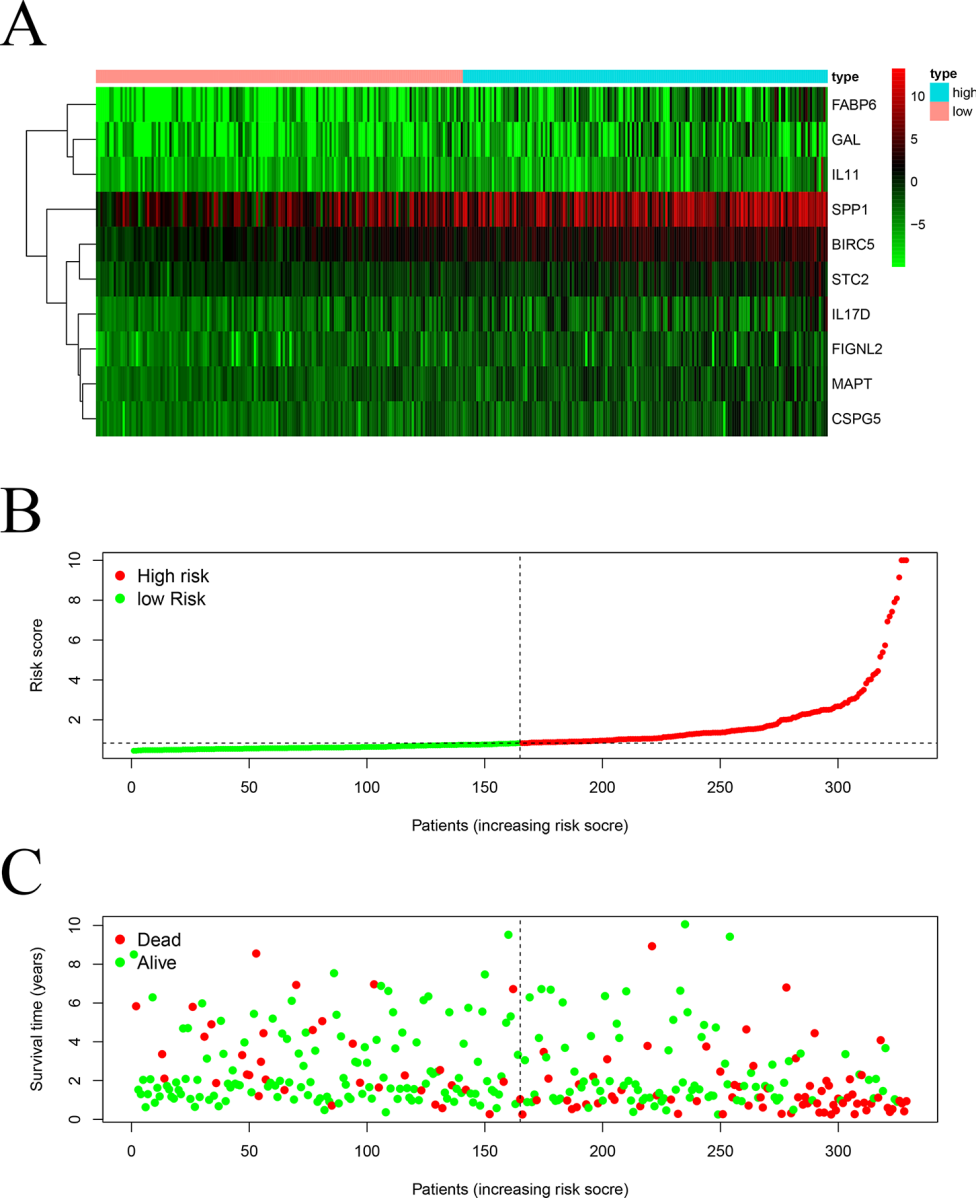
Construction of a TCGA prognostic model using survival-associated IRGs: Heatmap **(A)** of survival-associated IRGs in the prognostic model. **(B)** Rank of risk score and distribution of groups. **(C)** Survival status of patients in different groups.

We screened 22 survival-associated IRGs based on the overall survival (OS) of patients using univariate Cox regression analysis (**Figure 2**). *Via* multivariate Cox regression analyses of survival-associated IRGs text by AIC, we established a prognostic model that classified patients into high- and low-risk score groups based on OS using the median risk score as the cut-off (**Figure 3**).The survival risk score= (Expression level of *BIRC5* × 0.02296) + (Expression level of *CSPG5* × 0.33178) + (Expression level of *IL-11* × 0.01577) + (Expression level of *FABP6* × 0.07392) + (Expression level of *FIGNL2* × 0.44366) + (Expression level of *GAL* × 0.18222) + (Expression level of *IL17D* × 0.08771) + (Expression level of *MAPT* × 0.27133) + (Expression level of *SPP1* × 0.00015) + (Expression level of *STC2* × 0.02978).

By using GSE76427, we established a validation model that classified patients into high- and low-risk score groups based on OS using the median risk score as the cut-off (**Figure S1**).

### Functional Enrichment Analysis of DEIRGs

**Figure 4:**
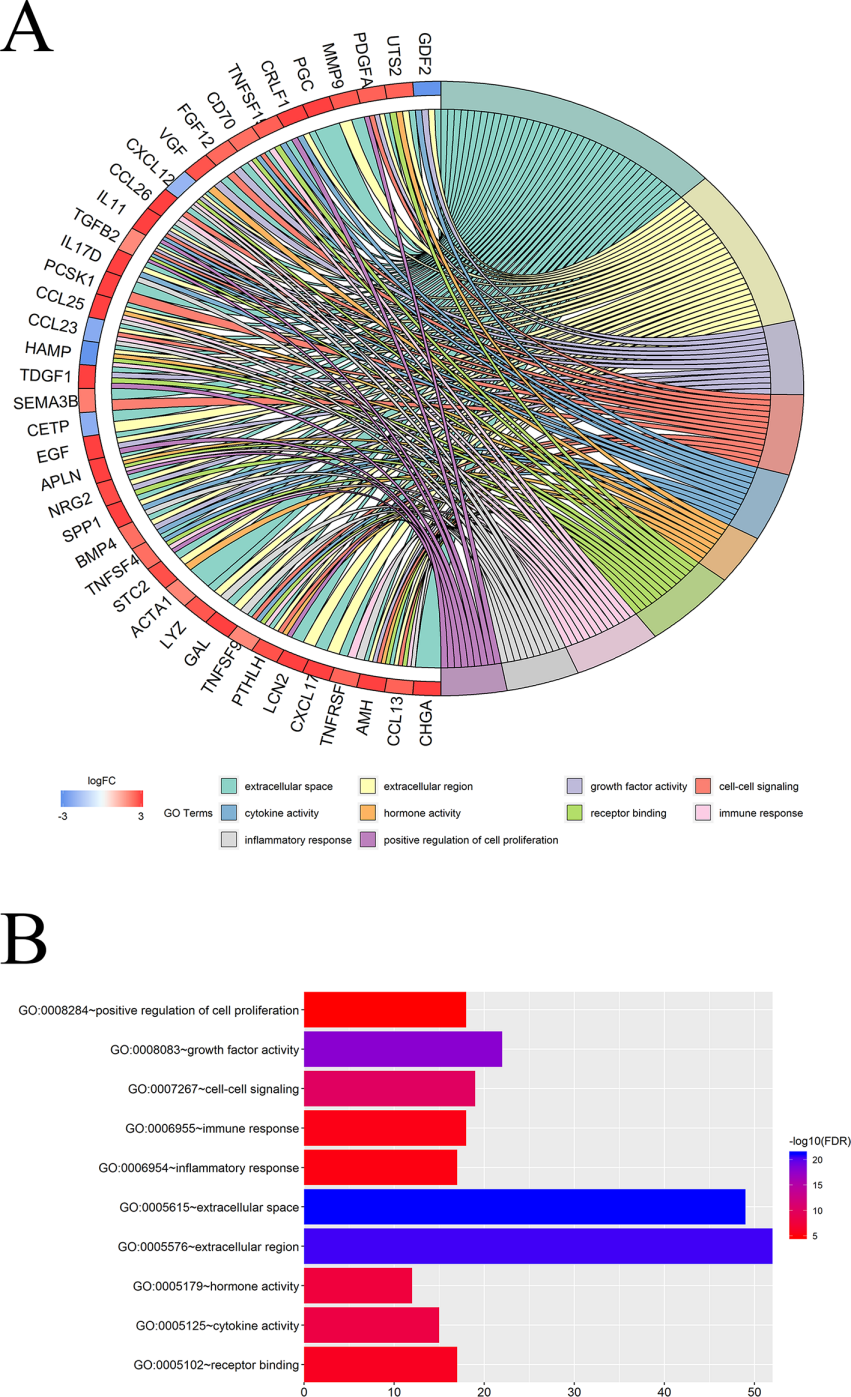
Functional enrichment analysis of DEIRGs: **(A, B)** GO biological process categories.

We further investigated the biological functions of 116 IRGs *via* GO annotation and KEGG pathway analyses. The obtained results showed that the extracellular region was the most frequent GO biological process category (*P* < 0.05). The top frequent, enriched GO networks are shown in **Figures 4A, B**.

**Figure 5:**
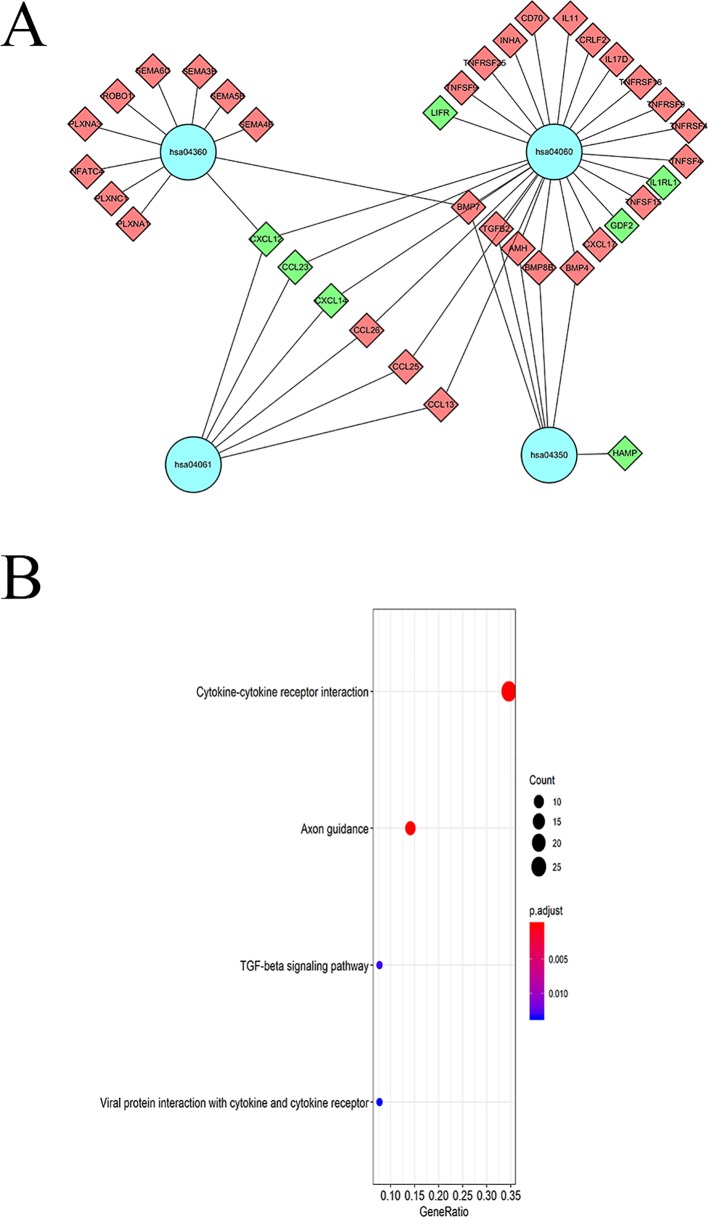
KEGG pathway analysis of DEIGRs: **(A)** Relationship between IRGs and KEGG pathways. **(B)** The significantly enriched KEGG pathway.

KEGG pathway analyses led to the identification of top significantly enriched pathways (**Figure 5A**); the “cytokine-cytokine receptor interaction” pathway was the most enriched one. Based on the relationship between IRGs and KEGG pathways, we constructed a network using Cytoscape (**Figure 5B**).

### DElncRNAs and DEmiRNAs in HCC

**Figure 6:**
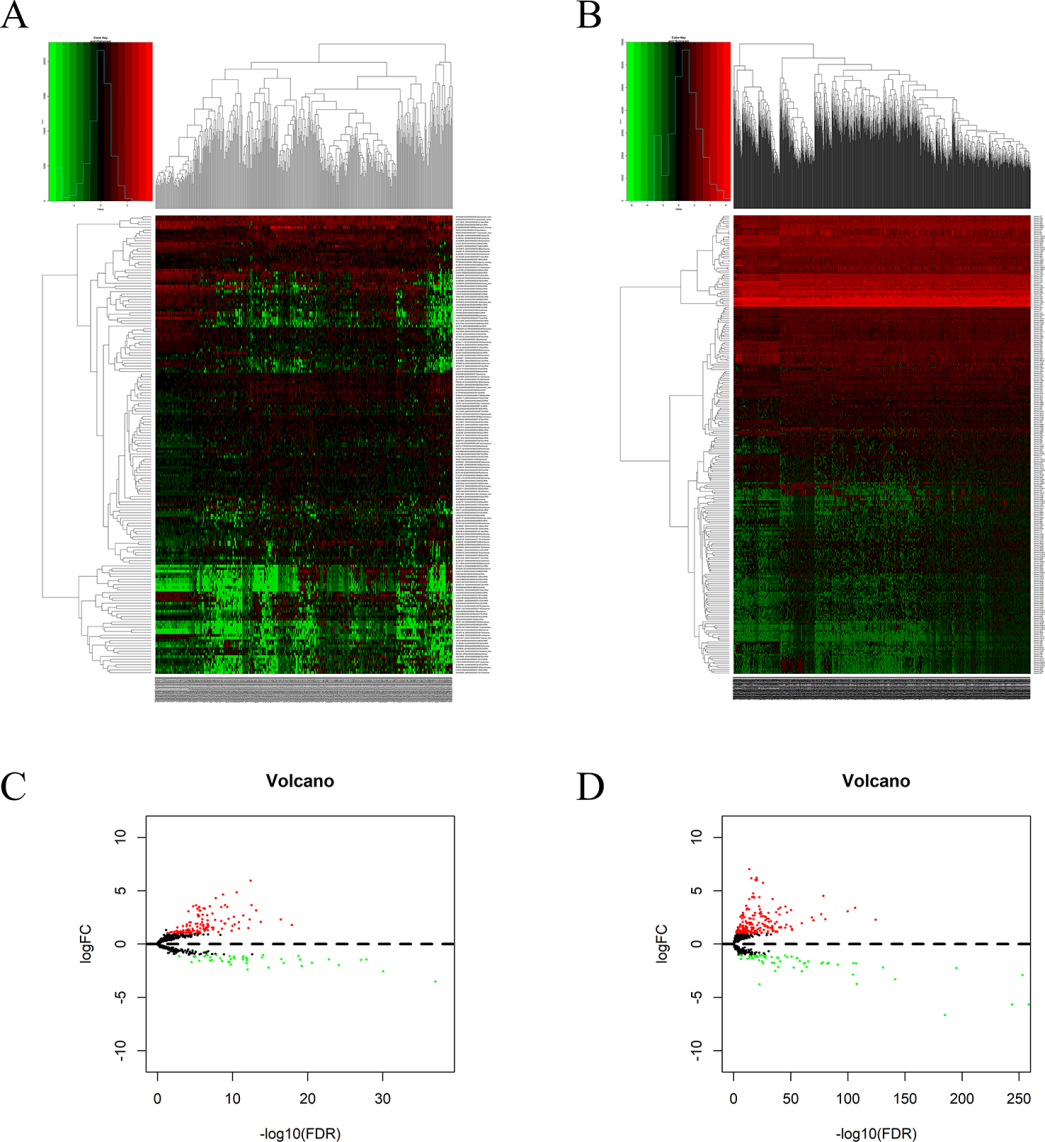
DElncRNAs and DEmiRNAs in patients with HCC: Heatmap **(A)** and volcano plot **(B)** of DElncRNAs in HCC and adjacent non-cancer liver tissues. Heatmap **(C)** and volcano plot **(D)** of DEmiRNAs in HCC and adjacent non-cancer liver tissues.

Significant DEGs were identified in 374 HCC and 50 adjacent non-cancer liver tissues from TCGA database by using the *edgeR* package in the R statistical environment. A total of 172 DElncRNAs (126 upregulated and 46 downregulated) and 251 DEmiRNAs (187 upregulated and 64 downregulated) were identified with the thresholds of |log_2_ FC| > 2.0 and adjusted *P* < 0.05. The distribution of DElncRNAs and DEmiRNAs was based on two parameters, FDR and logFC, and is represented as a volcano plot (**Figures 6A, C**). A heatmap was constructed to depict the expression data of DElncRNAs and DEmiRNAs (**Figures 6B, D**).

### Construction of the ceRNA Network

**Figure 7:**
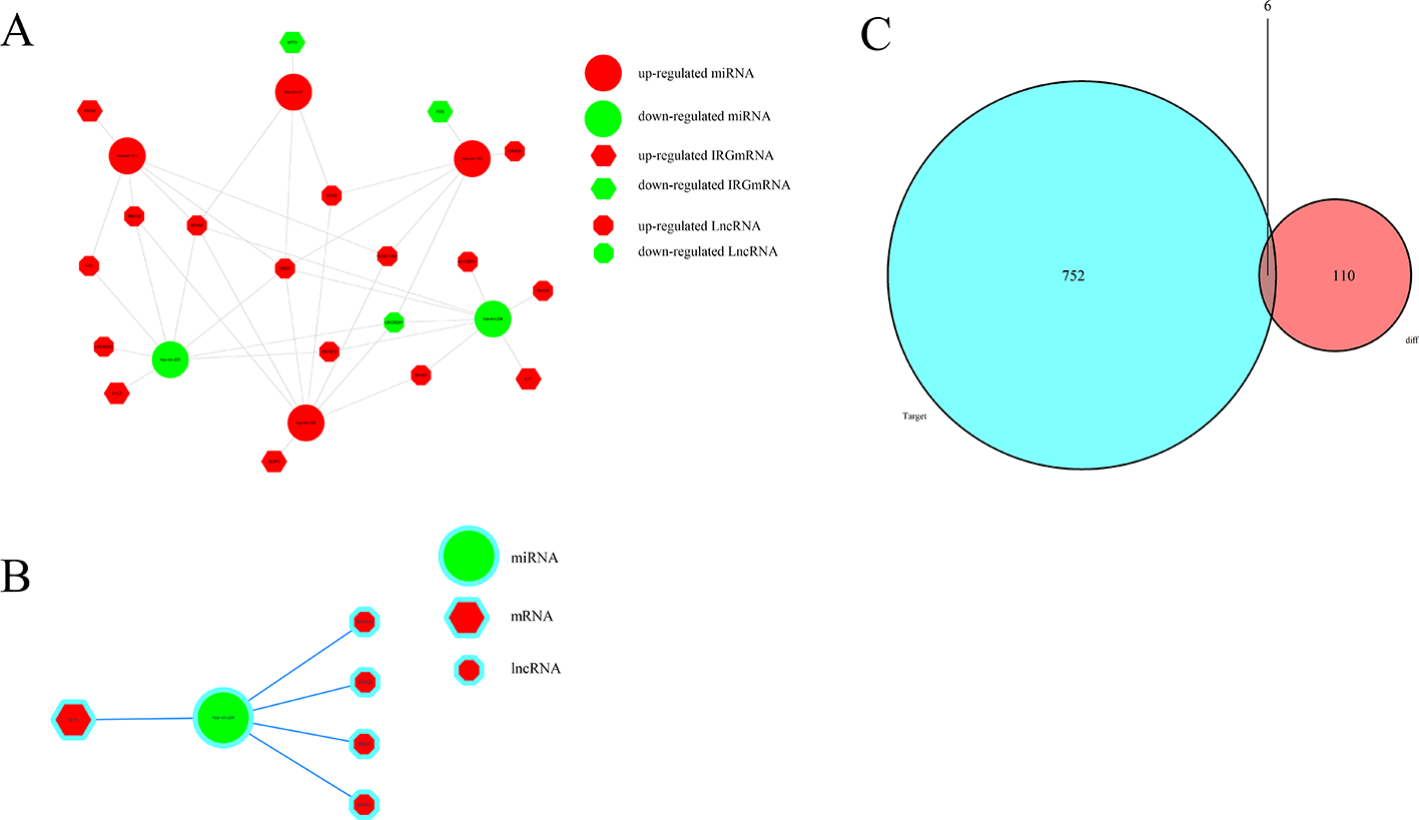
The ceRNA network of mRNA, miRNA, and lncRNA: **(A)** Relationship between survival-associated IRG mRNA, miRNA, and lncRNA. **(B)** IL-11/miRNA-204/LncRNA axis. **(C)** Venn diagram of DEmiRNA target mRNA and DEIRGmRNAs.

To further explore how lncRNAs mediate mRNA by combining miRNA in HCC, an lncRNA–miRNA–mRNA (ceRNA) network was constructed based on the above data. We used 172 DElncRNAs that were retrieved from the miRcode database and used the Perl program to identify 35 pairs of interacting lncRNAs and miRNAs. Moreover, miRTarBase, miRDB, and TargetScan databases were used to identify target mRNAs based on 251 miRNAs. The final target genes were selected, which were then included in all three datasets. Finally, six DEIRGmRNAs were included in the ceRNA network (**Figure 7C**). In total, 11 lncRNA nodes, six miRNA nodes, and six mRNA nodes were identified as having differential expression profiles in the ceRNA networks (**Figures 7A, B**).

### Construction of a PPI Network and Module Analyses

**Figure 8:**
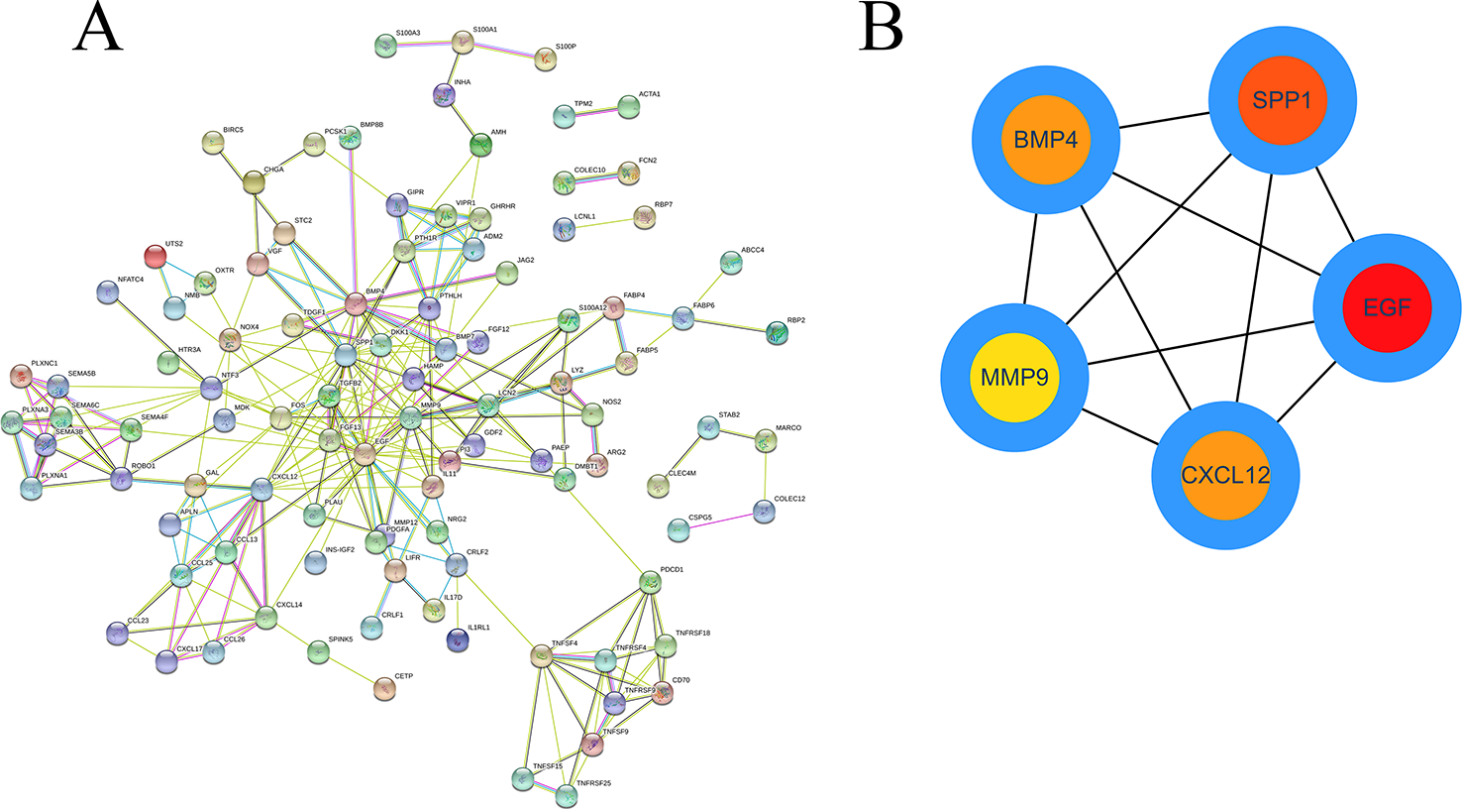
The PPI network of DEIRGs. **(A)** DEIRGs and **(B)** hub genes.

Based on the obtained DEIRGmRNAs, we used STRING to construct a PPI network; visualization was performed using Cytoscape. The Molecular Complex Detection plug-in for Cytoscape was then used to screen the modules of hub genes from the PPI network (**Figures 8A, B**).

### Clinical Outcome of Patients With HCC Using the IRGPM

**Figure 9:**
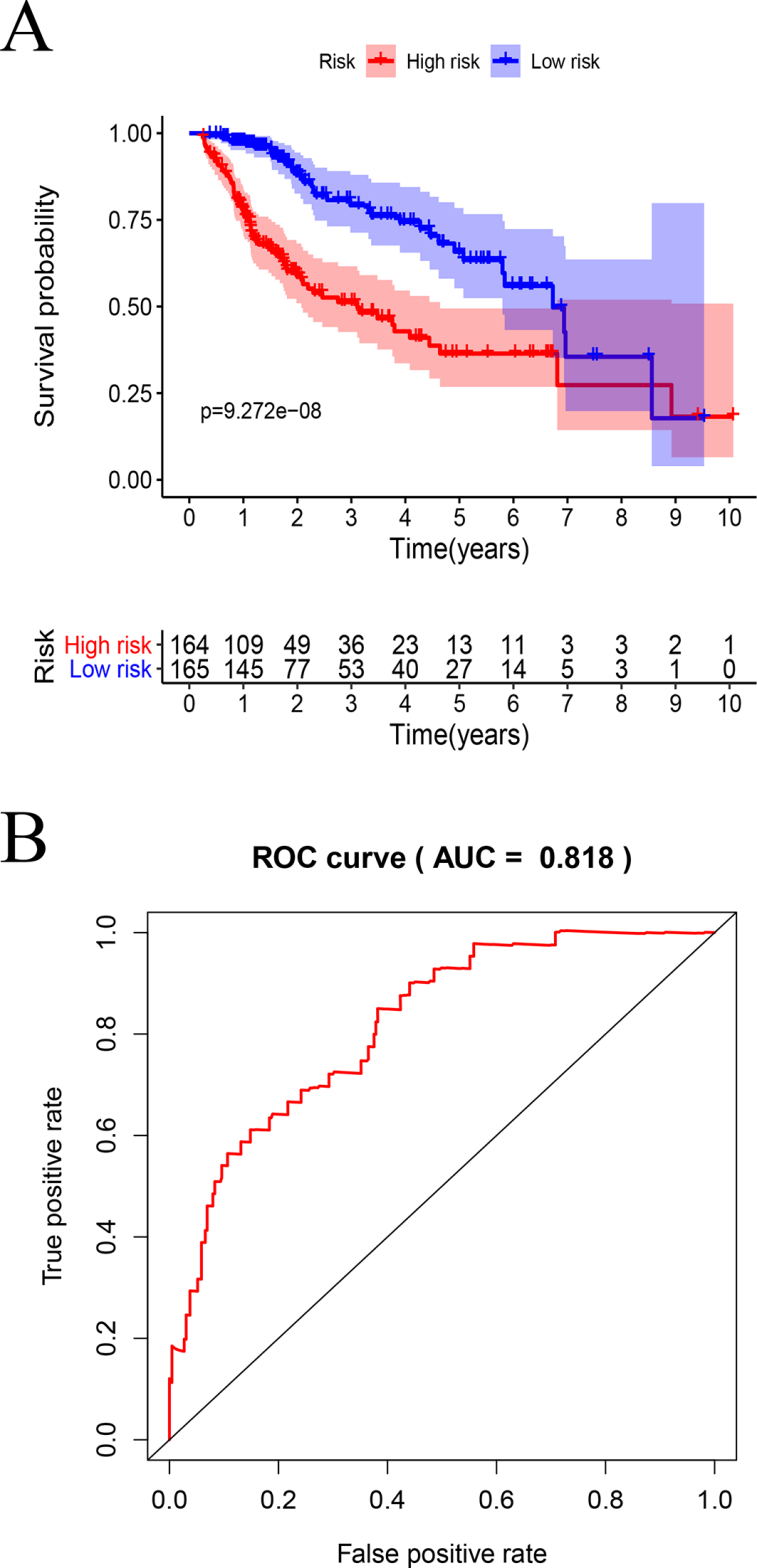
Survival analysis by the prognostic model: **(A)** Kaplan–Meier analysis using high- and low-risk score groups of patients with HCC. **(B)** ROC curve analysis.

By using KM curve, the prognostic of TCGA model (**Figure 9A**) and GSE76427 model (**Figure S2**) were analyzed.

**Figure 10:**
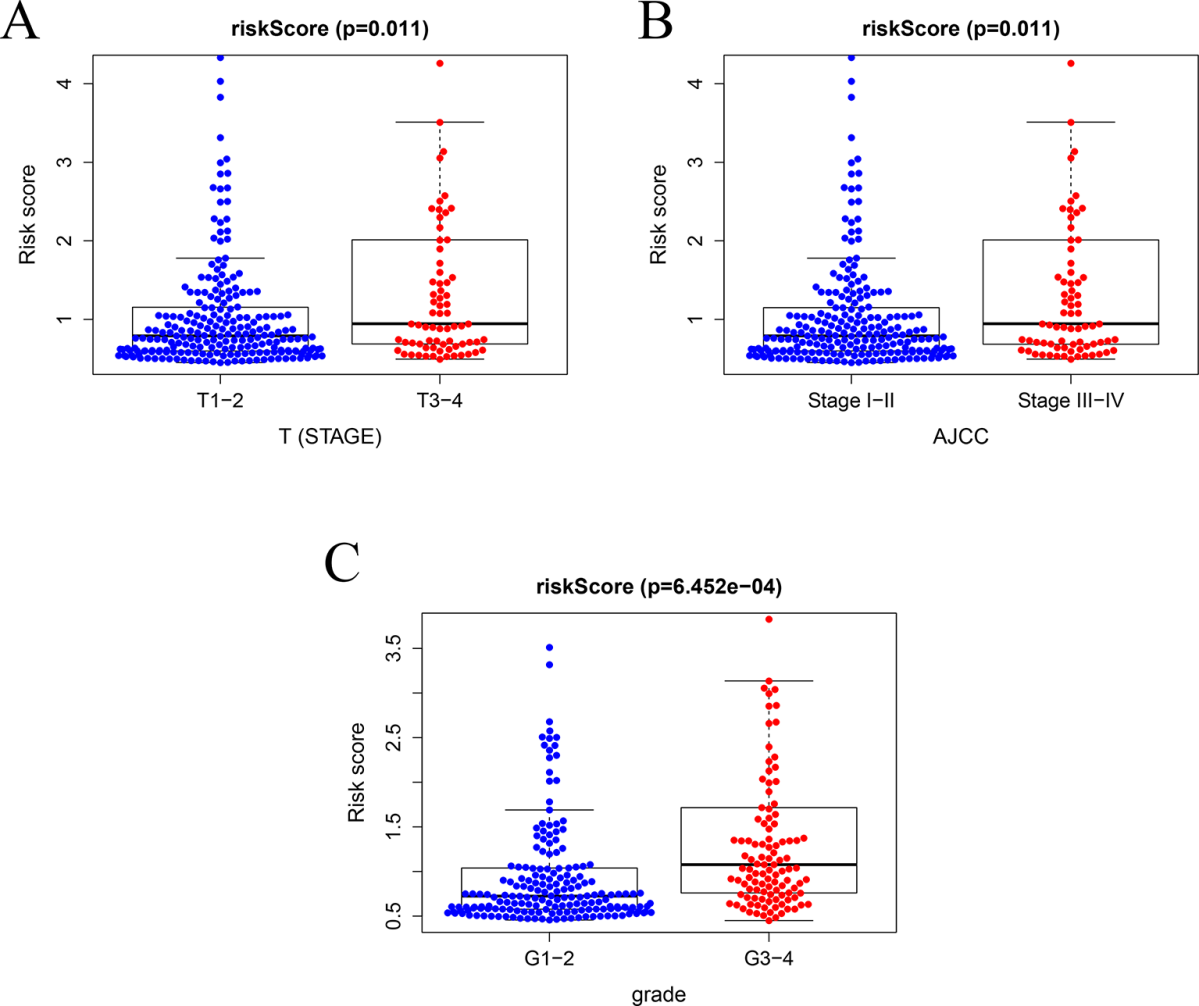
Relationships between risk score and clinical features. **(A)** Relationships between risk score and T stage, **(B)** between risk score and AJCC stage, and **(C)** between risk score and grade.

In TCGA model, the area under the ROC curve was 0.818, suggesting that the TCGA IRGPM could be used for monitoring survival (**Figure 9B**). Univariate analyses (**Table 3**) showed that high American Joint Committee on Cancer (AJCC) stage [hazard ratio (HR) = 1.866; 95% confidence interval (CI) = 1.488–2.341; *P* < 0.001), high T stage (HR = 1.826; 95% CI = 1.472–2.265; *P* < 0.001), and high risk score (HR = 2.238; 95% CI = 1.293-3.872; *P* < 0.001) were significant risk factors for poor prognoses. In the multivariate Cox regression analysis (**Table 2**), high risk score (HR = 2.071; 95% CI = 1.183–3.625; *P* < 0.001) was found to be independently associated with worse OS. The risk scores were significantly higher for patients with advanced T stage (**Figure 10A**), AJCC stage (**Figure 10B**), and grade (**Figure 10C**).

**Table 3:** Univariate and Multivariate Cox regression analysis of survival associated IRGS.

Risk Factors	Overall Survival (OS)			
	Univariate analysis		Multivariate analysis	
	HR (95% CI)	*P* value	OR (95% CI)	*P* value
Age	1.002(0.986−1.017	0.824	1.006(0.990−1.022)	0.484
gender	0.886(0.578−1.358)	0.580	1.110(0.713−1.729)	0.643
grade	1.173(0.886−1.551)	0.265	1.125(0.827−1.531)	0.452
AJCC stage	**1.866(1.488−2.341)**	<0.001**	1.247(0.461−3.378)	0.664
T stage	**1.826(1.472−2.265)**	<0.001**	1.408(0.541−3.666)	0.483
Risk score	**2.238 (1.293-3.872)**	<0.001**	**2.071** (**1.183**-**3.625**)	<0.001**

AJCC, American Joint Committee on Cancer; CI, confidence interval; HR, hazard ratio; IRGS, immune-related genes; OS, Overall Survival; T, tumor ** and bolded texts indicates P < 0.01 has statistically significant.

### Correlation Analysis of the IRGPM and Immune Cell Infiltration

**Figure 11:**
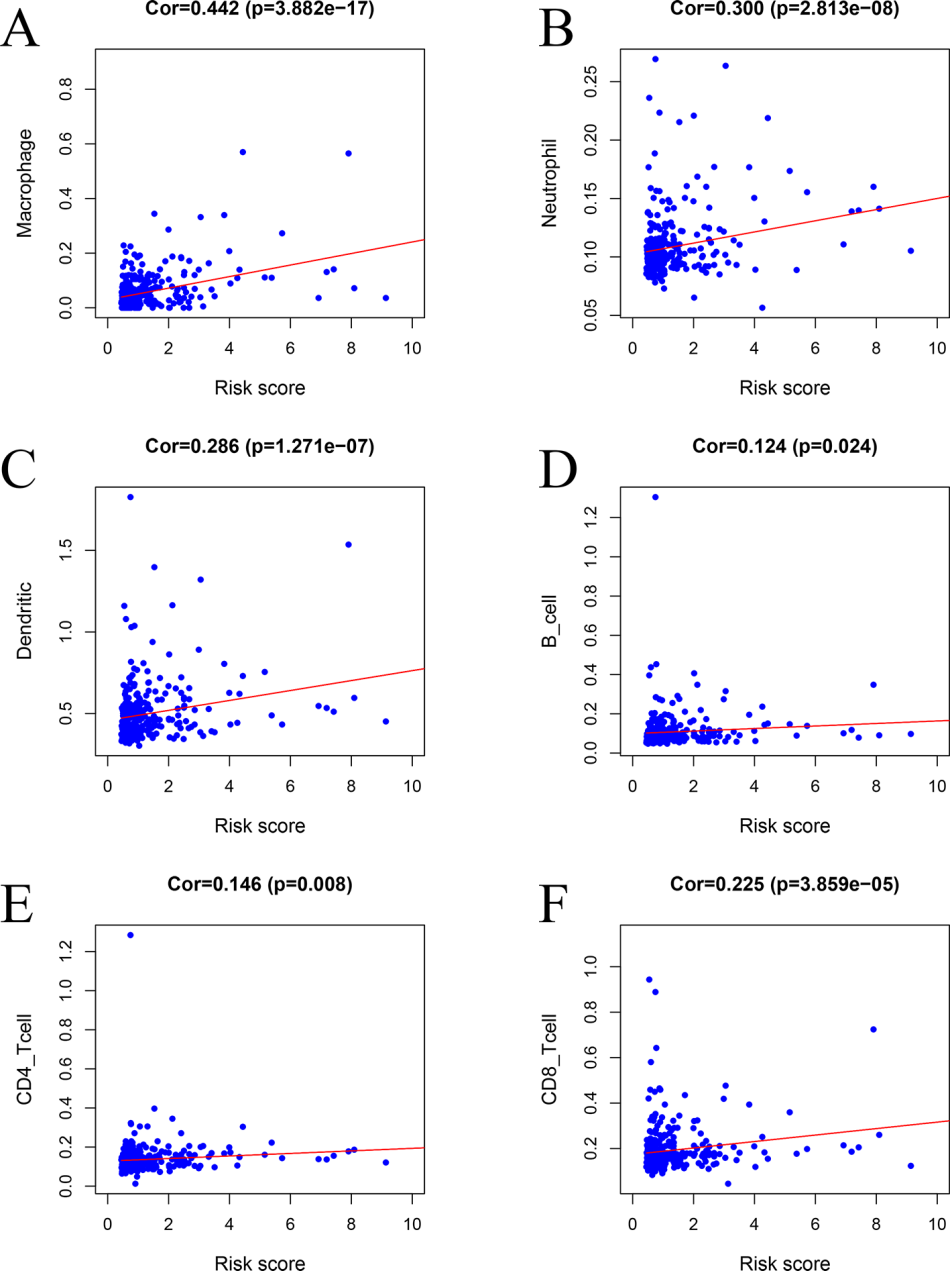
Relationships between the prognostic model and immune cell infiltration: **(A)** macrophages, **(B)** neutrophils, **(C)** dendritic cells, **(D)** B cells, **(E)** CD4 T cells, and **(F)** CD8 T cells.

The risk factors identified *via* the model were positively correlated with macrophages (*r* = 0.442, *P* < 0.001) (**Figure 11A**), neutrophils (*r* = 0.300, *P* < 0.001) (**Figure 11B**), DCs (*r* = 0.286, *P* < 0.001) (**Figure 11C**), B cells (*r* = 0.124, *P* = 0.024) (**Figure 11D**), CD4 T cells (*r* = 0.146, *P* = 0.008) (**Figure 11E**), and CD8 T cells (*r* = 0.225, *P* < 0.001) (**Figure 11F**).

### Analysis and Validation of Gene Expression

**Figure 12:**
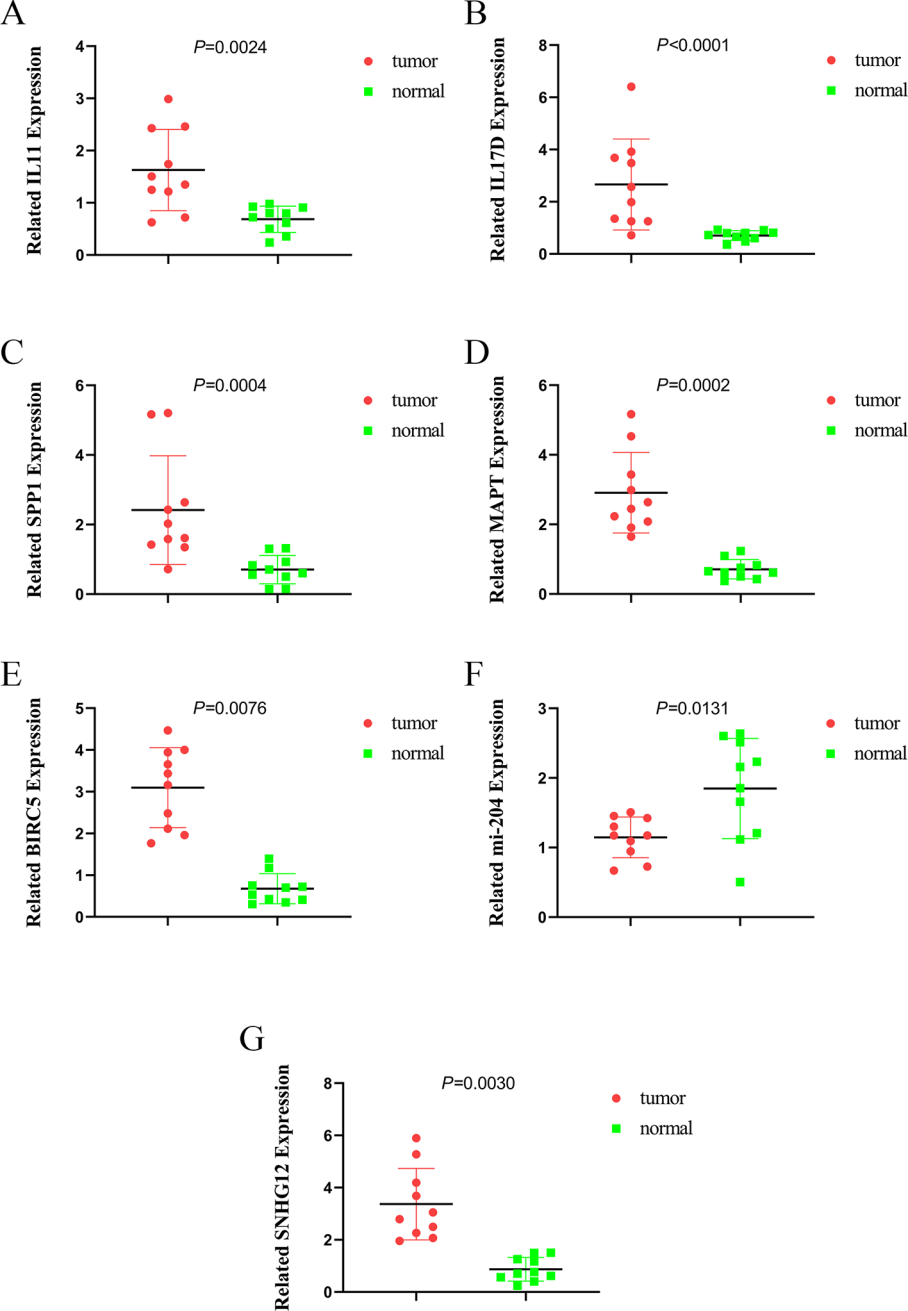
The expression of IL11, IL17D, SPP1, MAPT, BIRC5, miR-204, and SNHG12. **(A)** the expression of IL11, **(B)** the expression of IL17D, **(C)** the expression of SPP1, **(D)** the expression of MAPT, **(E)** the expression of BIRC5, **(F)** the expression of miR-204, and **(G)** the expression of SNHG12.

To further validate the expression of relevant key genes, mi-RNA and lncRNA in the prognostic model and ceRNA network, we randomly selected five mRNAs, one miRNA, and one lncRNA to measure the expression level use HCC tissue and adjacent normal tissues. We found that the expression is consistent with the TCGA database (**Figures 12A–G**).

## Discussion

Although the relationship between IRGs and tumor prognosis has been explored to a certain extent, that between IGRs and HCC still remains unknown. In the present study, we attempted to establish a prognostic model using IRGs in patients with HCC. Previous studies have indicated that IRGs are promising as prognostic indicators of early stage lung cancer. In a study conducted by (Li B. et al., 2017; Li T. et al., 2017) a prognostic immune signature of 25 gene pairs comprising 40 unique genes was created using the meta-training data set; the immune signature stratified patients with early-stage non-squamous non-small cell lung cancer into high- and low-risk groups in terms of OS, and remained as an independent prognostic factor in multivariate analyses (Li B. et al., 2017).

Through KEGG pathway analysis, we found that IRGs were mainly enriched in the “cytokine-cytokine receptor interaction” pathway. Cytokines secreted by various tumor cells have synergistic effects with immune cells in the tumor microenvironment (TME), resulting in increased tumor activity (de Looff et al., 2019). Macrophages and neutrophils can regulate the TRAIL/TRAIL-R system through cytokines to eliminate tumor cells (de Looff et al., 2019). Li et al. points out that the network between cytokines and tumor immunity has a bistable. This model has shown that tumors can use the bistable state to generate immunosuppression. If this eliminates this interaction, the immune system can return to immune boosting (Li and Levine, 2017). In addition, the authors have established various models to illustrate the interaction between tumor cells and macrophages in the tumor microenvironment, through which the stable state of tumor cells can be eliminated (Li et al., 2019). Two genes, i.e., IL-11 and IL-17, in the prognostic model were noted to be involved in this signaling pathway. Previous studies have indicated that cytokine signaling pathways are closely related to inflammation-mediated HCC (Hu et al., 2017). Inflammation plays a key role in liver cancer development (Capece et al., 2013). Chronic inflammation caused by HBV or HCV infection is a major factor in the development of HCC (Marrero et al., 2018). IL-17 is secreted by TH17 cells and is closely related to tumor growth (Miossec et al., 2009). Hu et al. indicated that IL-17 promotes the proliferation of liver cancer cells (HBV^+^) by activating the IL-6/STAT3 signaling pathway (Hu et al., 2017), and Tian et al. found that IL-17 expression and promoter methylation were closely related to the progression of chronic HBV infection, particularly in patients with HCC (Tian et al., 2019). Moreover, Wang et al. revealed that the IL-11 has a chief role in postsurgical HCC recurrence; they also found that the IL-11/STAT3 signaling pathway promotes the proliferation of tumor cells and that the inhibition of IL-11/STAT3 signaling could reduce the postsurgical recurrence of HCC (Wang et al., 2019). IL-11 regulates a variety of immune cells to participate in the immune response. For example, IL-11 can regulate macrophage function by inhibiting IL-1β (Schwertschlag et al., 1999). In addition, IL-11 can directly affect CD4T cells, promote the production of TH1 cells, and inhibit TH2 cells (Bozza et al., 2001; Curti et al., 2001).

To further identify the key genes participating in the prognostic model, we established PPI and ceRNA networks. In the PPI network, we screened five hub genes: EGF, SPP1, BMP4, MMP, and CXCL12. Three of these genes are associated with prognosis, and SPP1, also known as OPN, was one of the key genes for establishing the prognostic model. High expression levels of SPP1 can be reportedly detected in many tumors, including HCC (Gotoh et al., 2002; Reinholz et al., 2002; Urquidi et al., 2002). SPP1 has been reported to mediate macrophage polarization and induce immune escape in lung adenocarcinoma (Zhang et al., 2017). By activating the PI3K/Akt signaling pathway, OPN can cause HCC metastasis (Yu et al., 2018).

In case of the ceRNA network, we used DEmiRNAs and DElncRNAs to construct an lncRNA–miRNA–mRNA network. Salmena et al. proposed that all types of RNAs compete with each other for miRNAs, resulting in large-scale *trans*-regulatory crosstalk across the transcriptome as a whole (Salmena et al., 2011). In the ceRNA network, we identified that the expression of IL-11 was upregulated whereas that of miRNA and SHNG12 was upregulated. Several studies have reported that miRNA-204 expression is downregulated in tumor tissues than in normal tissues, and it is thus generally regarded to be a tumor suppressor gene (Yin et al., 2014; Liu et al., 2015; Xia et al., 2015; Hong et al., 2019). Ge et al. indicated that miRNA-204 can attenuate the proliferation of HCC cells by inhibiting the lncRNA HOTTIP (Ge et al., 2015). Lan et al. found that the expression levels of SNHG12 is higher in HCC compared with adjacent normal tissues, SNHG12 can promote the proliferation and metastasis of HCC through target miR-199 (Lan et al., 2017). Therefore, we hypothesized that IL-11 and SHNG12 competitively bind to miRNA204, resulting in the downregulation of miRNA204 expression with impaired anticancer effects, *via* further promoting the occurrence and development of HCC.

In the past decade, liver cancer treatment has shown marginal improvement, particularly for patients who are inoperable or at stage IV. Sorafenib has been reported to prolong the OS of patients with advanced HCC in the Asia-Pacific region (Cheng et al., 2009), and a study reported the median survival time for lenvatinib was 13.6 months, being non-inferior to sorafenib (12.3 months), in patients with advanced HCC (Kudo et al., 2018). In the past decade, the emergence of immunotherapy has revolutionized cancer treatment, and immunological checkpoint inhibitors appear promising for treating patients with liver cancer (El-Khoueiry et al., 2017; Zhu et al., 2018).

A recent study proposed that the functional role of innate lymphoid cells in antitumor immunity is complex (Gajewski et al., 2013). miRNAs in the tumor microenvironment have been reported to have a crucial role in the development of cancer and its progression (Kuninty et al., 2016). Interestingly, in this study, we found that our prognostic model was positively correlated with the infiltration of six subtypes of tumor-infiltrating immune cells.

Tumor-associated macrophages are the major components of tumor inflammatory infiltration and are key mediators between tumors and inflammation (Mantovani et al., 1992). Guo et al. found that M2 macrophages in the HCC microenvironment can secrete large amounts of IL-17 and inhibit oxaliplatin-induced apoptosis by activating chaperone-mediated autophagy (Guo B. et al., 2017). Tumor-associated neutrophils are also an important part of the tumor microenvironment and can promote various biological activities of tumors (Galdiero et al., 2013). Neutrophils can secrete proinflammatory and immunoregulatory factors, such as neutrophil elastase, hepatocyte growth factor, and β2-integrins, which have paracrine effects on tumor biology (Wislez et al., 2003; Houghton et al., 2010; Strell et al., 2010). Tumor-associated neutrophils/macrophages can reportedly promote the survival and proliferation of malignant tumors; promote the remodeling of extracellular matrix, blood vessels, lymphangiogenesis, and invasion; and also facilitate the transfer of adaptive immunity (Mantovani et al., 2008; Qian and Pollard, 2010; Mantovani et al., 2011).

Zhou et al. indicated that intratumoral infiltration of plasmacytoid DCs is a novel indicator of poor prognosis in patients with HCC. They also reported that an increase in intratumoral plasmacytoid DCs was associated with increased intratumoral infiltration of Foxp3^+^ regulatory T (Treg) cells and IL-17-producing cells (Zhou et al., 2019). Tumor-infiltrating B lymphocytes evidently play a dual role in some types of cancers: they combat cancer growth *via* antigen-restricted tumoricidal immune response or promote tumor progression *via* the induction of immune suppression (Guo S. et al., 2017). Another study reported that patients with HCC exhibited markedly higher levels of IL-10-expressing B cells as compared to healthy controls (Xue et al., 2016).

T cells have many subpopulations, including cytotoxic T cells (CTL), helper T (TH) cells, and Treg cells. Numerous previous studies have confirmed that CD4^+^CTLs or CD8^+^CTLs can inhibit the proliferation of HCC cells (Fu et al., 2013; Yao et al., 2017). Treg cells have a distinct ability to evade immune suppression to impair antitumor ability, helping tumor cells to escape immune surveillance (Du et al., 2012). Previous studies have indicated that high levels of CD4^+^CD25^+^Treg cells are positively correlated with the expression of HBV DNA in the serum of patients with chronic HBV infection, and antiviral therapy can reduce the frequency of Treg cells (Yan et al., 2014). Zhou et al. demonstrated that tumor-associated neutrophils can recruit Treg cells and macrophages, leading to progression of HCC and promoting resistance to sorafenib (Zhou et al., 2016). The complexity of the tumor microenvironment and immune system makes the response to treatment difficult to predict; thus, personalized treatment of tumors is imminent.

This study has some limitations that should be borne in mind in interpreting the findings. Firstly, the model established by us showed a positive correlation with the infiltration of T cells and DCs. We know that T cells and DCs have different subtypes. However, a progressive deficit in CD4^+^ CTLs induced by increased FoxP3^+^ Treg cells is reportedly correlated with poor survival and high recurrence rates in patients with HCC (Fu et al., 2013). We propose that subpopulations of cells should be individually studied in future studies. Finally, we identified some key genes and also explained their potential mechanisms; however, further studies on these are warranted.

In summary, we used survival-associated IRGs to establish a prognostic model. We identified some key genes *via* the PPI and ceRNA networks and that the expression of IL-11 was upregulated whereas that of miRNA and LINC00261 was downregulated; moreover, the model was positively correlated with the infiltration of several subtypes of tumor-infiltrating immune cells. The prognostic model was also related to clinical characteristics such as AJCC stage, T stage, and HCC differentiation. We believe that the model provides sufficient information for prognosis prediction and immunotherapy of patients with HCC.

## Data Availability Statement

The datasets analysed for this study were obtained from TCGA data portal (https://portal.gdc.cancer.gov/) and GEO data portal (https://www.ncbi.nlm.nih.gov/geo/).

## Ethics Statement

The studies involving human participants were reviewed and approved by Medical Ethics Committees of the Affiliated Suzhou Hospital of Nanjing Medical University. The patients/participants provided their written informed consent to participate in this study.

## Author Contributions

Z-LD and Y-QH contributed toward the conception and design of this study. JZ and J-JW organized the database. WS performed statistical analyses. HW, W-JW, and T-YH wrote the first draft of the manuscript. T-YH and WS wrote sections of the manuscript. All authors contributed to manuscript revision and have read and approved the submitted version.

## Funding

This study was supported by the Science and Education for Health Foundation of Suzhou for Youth (grant no. KJXW2018030 and KJXW2018032), the Science and Technology Project Foundation of Suzhou (grant no. SS201651), Suzhou Oncology Clinical Center (grant no. Szzx201506), Project of Clinical Expert Team Introduction in Suzhou (grant no. SZYJTD201807), Project of science and technology development plan in Suzhou (grant no. SYSD2018138) and the Jiangsu Province Medical key discipline (grant no. ZDXKC2016007).

## Conflict of Interest

The authors declare that the research was conducted in the absence of any commercial or financial relationships that could be construed as a potential conflict of interest.

## Supplementary Material

Supplementary Figure S1 Construction of a validation model of GSE76427. Heatmap **(A)** of survival-associated IRGs in the prognostic model. **(B)** Rank of risk score and distribution of groups. **(C)** Survival status of patients in different groups.

Supplementary Figure S2 The prognosis of validation model.
