# Supplementary figures and images for: Establishment of a Prognostic Model Using Immune-Related Genes in Patients With Hepatocellular Carcinoma
Source: Front Genet. 2020 Feb 25;11:55. doi: 10.3389/fgene.2020.00055 (PMC7052339; doi:10.3389/fgene.2020.00055)

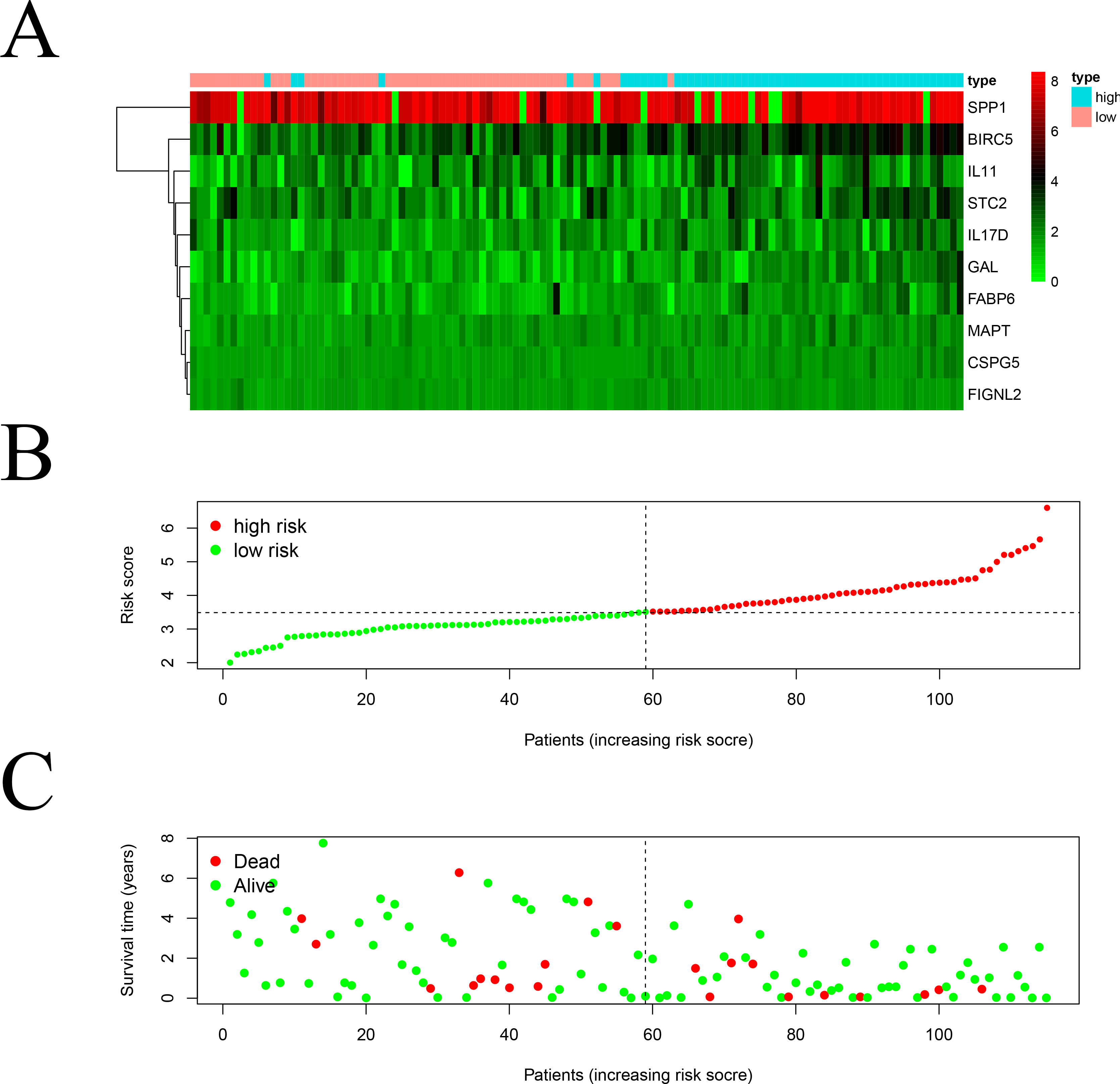

Supplement: Supplementary Figure S1 — Construction of a validation model of GSE76427. Heatmap (A) of survival-associated IRGs in the prognostic model. (B) Rank of risk score and distribution of groups. (C) Survival status of patients in different groups. [file Image_1.tif]

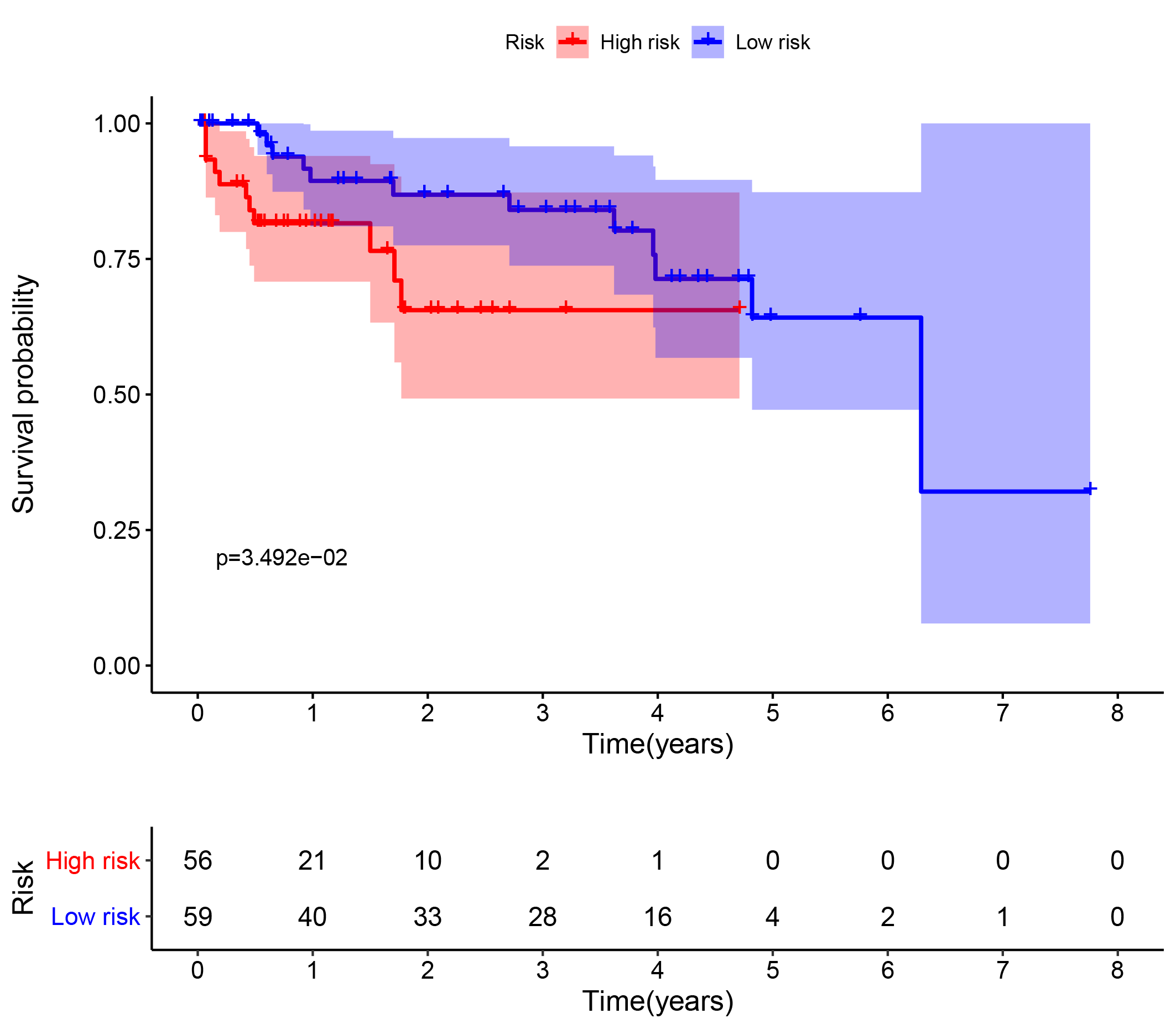

Supplement: Supplementary Figure S2 — The prognosis of validation model. [file Image_2.tif]
