# Supplementary material for: Establishment of a Prognostic Model Using Immune-Related Genes in Patients With Hepatocellular Carcinoma
Source: Front Genet. 2020 Feb 25;11:55. doi: 10.3389/fgene.2020.00055 (PMC7052339; doi:10.3389/fgene.2020.00055)
Supplement: Supplementary file 3 [file Table_1.docx]

| clinical features |  |  |
| --- | --- | --- |
| Age (years) | media | 64 |
|  | Rage | 29-93 |
|  | Numbers of patients (n=115) | Numbers of patients (%) |
| Gender | Female | 22 (19.13) |
|  | Male | 93 (80.87) |
| AJCC stage | I | 55 (47.83) |
|  | II | 35 (30.43) |
|  | III | 21 (18.20) |
|  | IV | 4 (3.54) |

**Table S1. The clinical features of GSE76427 hepatocellular carcinoma patients**

**Abbreviation:** AJCC, American Joint Committee on Cancer.
